# Merging citizen science with epidemiology: design of a prospective feasibility study of health events and air pollution in Cologne, Germany

**DOI:** 10.1186/s40814-023-01250-0

**Published:** 2023-02-22

**Authors:** Sara-Marie Soja, Robert Wegener, Natalie Kille, Stefanie Castell

**Affiliations:** 1 Department for Epidemiology, Helmholtz Centre for Infection Research, Inhoffenstr. 7, Brunswick, Lower Saxony 38124 Germany; 2 Forschungszentrum Jülich, Institute for Energy and Climate Research, IEK-8: Troposphere, Wilhelm-Johnen-Straße, Jülich, North Rhine-Westphalia 52428 Germany; 3 German Centre for Infection Research (DZIF), Inhoffenstr. 7, Brunswick, Lower Saxony 38124 Germany

**Keywords:** Citizen science, Acute respiratory infections, COVID-19, Feasibility study, Air pollution, Symptom monitoring, m/eHealth, eResearch, Low-cost sensor, Sensor network

## Abstract

**Background:**

Citizen science as an approach to merge society and science is not a new paradigm. Yet it is not common in public health, epidemiology, or medical sciences. SMARAGD (Sensors for Measuring Aerosols and ReActive Gases to Deduce health effects) assesses air pollution at participants’ homes or workplaces in Cologne, Germany, as feasibility study with a citizen science approach. Personal exposure to air pollutants is difficult to study, because the distribution of pollutants is heterogeneous, especially in urban areas. Targeted data collection allows to establish connections between air pollutant concentration and the health of the study population. Air pollution is among the most urgent health risks worldwide. Yet links of individualized pollution levels and respiratory infections remain to be validated, which also applies for the feasibility of the citizen science approach for epidemiological studies.

**Methods:**

We co-designed a prospective feasibility study with two groups of volunteers from Cologne, Germany. These citizen scientists and researchers determined that low-cost air-quality sensors (hereafter low-cost sensors) were to be mounted at participants’ homes/workplaces to acquire stationary data. The advantage of deploying low-cost sensors is the achievable physical proximity to the participants providing health data. Recruitment started in March 2021 and is currently ongoing (as of 09/22). Sensor units specifically developed for this study using commercially available electronic sensor components will measure particulate matter and trace gases such as ozone, nitrogen oxides, and carbon monoxide. Health data are collected using the eResearch system “Prospective Management and Monitoring-App” (PIA). Due to the ongoing SARS-CoV-2 pandemic, we also focus on COVID-19 as respiratory infection.

**Discussion:**

Citizen science offers many benefits for science in general but also for epidemiological studies. It provides scientific information to society, enables scientific thinking in critical discourses, can counter anti-scientific ideologies, and takes into account the interests of society. However, it poses many challenges, as it requires extensive resources from researchers and society and can raise concerns regarding data protection and methodological challenges such as selection bias.

## Background

Epidemiology is a term now familiar to most people due to the current coronavirus pandemic. Newspapers and online media report regularly on topics of epidemiology or the work of epidemiologists [1–3]. Citizen science seems to be less familiar [4, 5]. Citizen science is not a science per se but an approach to join citizens and researchers to do science together [6]. Such projects excel at facilitating inclusion of citizens in science, leading to relevant and meaningful results that benefit both science and society [6]. Citizen science has gained popularity in recent years also due to the availability of digital communication devices [7]. Citizen science, however, is not yet well-established in all research areas. So far, it is a rather well-known approach in life sciences or environmental sciences, whereas it is less common in medical research fields, like public health and epidemiology [8–10]. For example, as of September 2022, there are 540 hits for the search term “citizen science” in the SciFinder database with the context of public health, biomedical research, and medicine but 810 for environmental science, environmental monitoring, and biology [11]. On the Web of Science database, there are 234 hits for the search term “citizen science” and “public health”, “biomedical research”, and “medicine”, whereas 436 hits were retrieved for the combination of “citizen science” and “environmental sciences”, “environmental monitoring”, and “biology” [12]. This lack of integration into medical and related research prevents benefits coming into effect, such as empowerment and increased self-efficacy of participants, as well as meaningful prioritization of research areas in the eyes of citizens and society [10, 13]. This absence of use might stem from challenges with data security and ethics [10, 14–16].

### The study SMARAGD

**Fig. 1:**
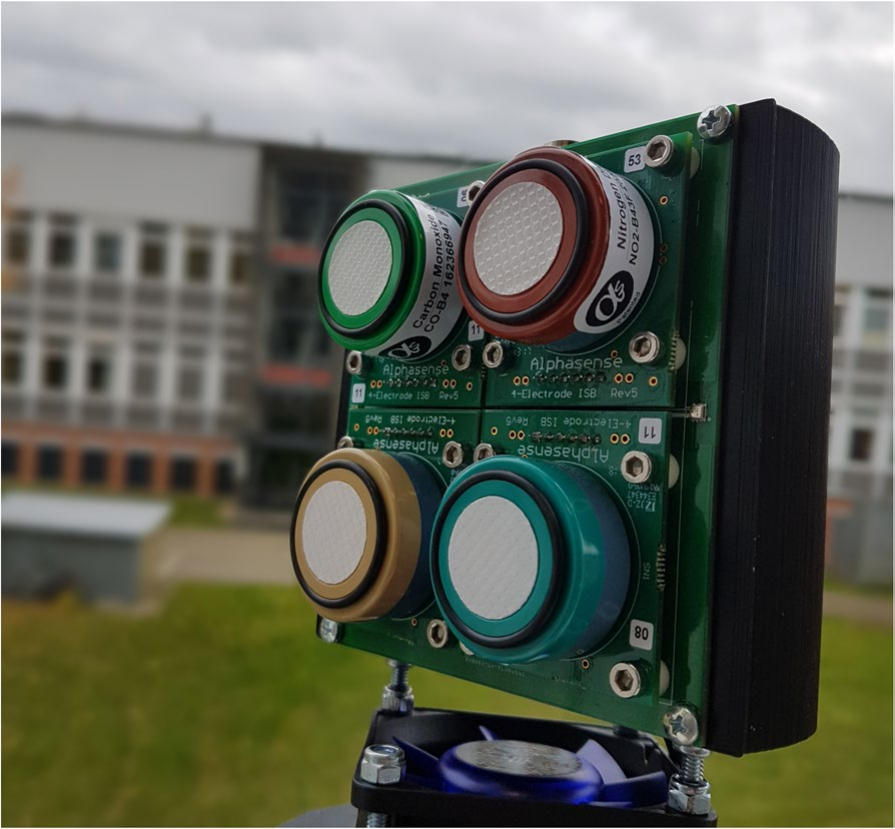
Low-cost air quality sensors with the weather protection removed. The picture shows the front side with the four gas sensors

Our project “Sensors for Measuring Aerosols and ReActive Gases to Deduce health effects” (SMARAGD) is a pilot study. SMARAGD utilizes collection of a unique combination of targeted air pollution data with time-sensitive infection data provided weekly by participants. Air pollutants are often very heterogeneously distributed with traffic-related emissions varying on the scale of 100 m, but they are hardly measured apart from monitoring stations of the official networks that are often deployed at coarse spatial scales, which fail to capture all relevant heterogeneity [17, 18]. To address this, we measure air pollutants of interest using specific low-cost sensors (Fig. 1) that can be placed at citizens’ place of residence or workplace. The study is based on a cooperation between the Helmholtz Centre for Infection Research (HZI), the Forschungszentrum Jülich (FZJ), and the Open-Knowledge Lab Cologne (OK Lab) to explore associations between air pollution and respiratory infections in Cologne, Germany. The Helmholtz Centre Munich (HMGU) is also involved as an additional partner to provide background information for the project’s website. As funding of a pilot study is limited, the cohort size of this study has to be restricted. Therefore, there will be limitations in the precision of the estimate of the association between pollution and health. Due to the innovative and unique nature of the project, we also focus on the evaluation of the feasibility of such a study as citizen science project.

The World Health Organization (WHO) has declared air pollution to be one of the greatest risks for deteriorating health and premature death, especially for low- and middle-income countries, but also for high-income countries [19, 20]. Even though air pollution is decreasing overall in Germany, it still peaks in urban areas [21] and varies greatly depending on individual circumstances [18]. Air pollution affects many systems like the lungs or the cardiovascular system [22, 23]. The extent of the impact of air pollution on respiratory infections is a matter of scientific debate. Research often focusses on indoor air pollution [24–28] or specifically on lower-respiratory-tract infections [26, 29, 30]. Low-cost sensors to measure air pollution are a relatively new tool [31–34], and to our knowledge, no prior study has directly attempted to link their data to the people’s health. Previous studies linking health and air pollution [35, 36] have used data products from the EURAD (EURopean Air pollution Dispersion) model [37] with a spatial resolution of 1 × 1 km^2^. However, it is known that air pollution can vary on spatial scales smaller than this [17]. The value of individualized air pollution data, measured where participants spend a large proportion of their time, is not yet recognized in epidemiological research. Due to the ongoing pandemic, COVID-19 came into focus as health outcome as well. The eResearch system PIA (Prospective Monitoring and Management App) is used to measure symptoms and health-related factors [38, 39].

### Aims and objectives

Our primary aim is to conduct a feasibility study that describes air pollution data gathered at participants’ place of residence or workplace and data on participants’ symptoms of acute respiratory infections, including COVID-19. We will explore associations between the amount of air pollution measured locally and respiratory infections. One secondary aim of SMARAGD is to evaluate the feasibility of the citizen science approach in epidemiological studies. For this, we collect data on compliance with app use and satisfaction and evaluate data quality. We presume that such parameters differ from standard epidemiological cohort studies. In a citizen science study, there is a closer relationship of study participants, researchers, and the research topics, and interests and concerns of citizens, especially those affected in areas with potentially high concentrations of air pollutants, are taken into account. Regarding feasibility of our study approach, the performance of the low-cost sensors in the project will be evaluated against standard air pollution measurements.

Thirdly, due to SMARAGD’s nature as citizen science project, we also collect reports on exposures like stress, sleep, vaccination, life quality, and adherence to pandemic non-pharmaceutical interventions to be used as control variables in the main analysis but also for explorative analysis regarding effects on symptoms of acute respiratory infections.

Generally, our goal is to enhance knowledge transfer from citizens to researchers and from researchers to citizens making research more accessible and relevant to all stakeholders.

## Methods/design

### Study design

SMARAGD is a prospective cohort study with a citizen science approach based in Cologne, Germany, similar to a population-based study. One difference to a classical epidemiological observational cohort study is that the participants consist of a convenience sample, i.e., the citizen scientists, rather than a random sample from the population of interest. Moreover, we designed the project with the help of the members of the OK Lab Cologne and the Cologne School of Artificial Intelligence (AI). Both are groups of people that work as volunteers in their spare time on open-source software and open data [40, 41]. Since there were already air quality monitoring activities in these groups, we established a working relationship. While the overall topic of the study was predetermined with the funding application, the exact scope of the project was intended to be collaboratively decided between citizens and researchers. Therefore, we conducted 14 workshops from November 2019 to November 2021 for and with citizen scientists in which they participated passively and actively, sometimes under guidance. Here, active participation refers to steps such as cognitive pretesting of questionnaires, whereas passive participation refers to the exchange of information on the project and the discussion of further steps. According to Frederking et al. [9], this corresponds to a level of citizen participation of collaboration, co-production, and co-creation. We did not reach the level of cooperation described by Frederking et al., as the overall topic was determined without citizen scientists.

In addition, HMGU provided content for a study-specific website to deliver high-quality information on air pollutants and their impacts on human health [42, 43]. The topics hosted by the website were decided during the first workshops and are based on questions of the citizen scientists. We planned the field phase with both sensors and the eResearch app PIA for 1 year to cover all four seasons.

### Low-cost air pollution sensors

For SMARAGD, we developed multi-sensor instruments for measurements of the air pollutants of interest; both trace gases and particulate matter (PM). We measure the trace gases such as carbon monoxide (CO), nitrogen oxides (NO, NO_2_), and ozone (O_3_) by electrochemical sensors that are commercially available from Alphasense (part number: CO-B4, NO-B4, NO2-B43F, OX-B431, respectively). PM_2.5_ and PM_10_ (PM with sizes up to 2.5 and 10 μm) are quantified by laser scattering using the NOVA SDS011 sensor. Additionally, we collect data on temperature and relative humidity to correct the low-cost sensor data for cross-sensitivities. We selected these sensors to stay in the proposed budget while achieving a good reliability and robustness for ambient air measurements over the project’s duration. Through a collaboration with the Cologne-based company Press Every Key (Cologne, Germany), 100 self-contained weatherproof nodes were produced for SMARAGD. The pure material costs amounted to 392 € per sensor node. We characterized the low-cost sensors against reference instrumentation in a controlled laboratory environment at FZJ. FZJ will distribute the instruments to the participants. The participants will install the sensors outside of their respective residence or workplace. Once configured, the instruments will automatically measure and send data to a server hosted by FZJ.

### PIA — prospective monitoring and management app

PIA is a free and open-source eResearch system developed since 2017 by the Department for Epidemiology at the HZI [38, 39, 44]. It includes a survey tool specifically designed for implementation in cohort studies. PIA displays questionnaires for participants and can be used flexibly via a mobile or a web app. It allows timely data collection and aims to increase response of compliance compared to paper-based studies. Additionally, PIA allows integration and management of samples of biospecimens [39]. The system is already in use in several epidemiological studies [45].

### Study population

Adults living in Cologne who are willing to use the eResearch system PIA on either a smartphone, tablet, or computer, who are able to speak and read German and are willing to operate an air pollution sensor or live in a household/work with someone who is willing to do so, are eligible to participate. We do not exclude vulnerable groups such as pregnant, breastfeeding women, or elderly people, as no intervention is part of this study. All participants complete an informed consent form before receiving any questionnaires. Initially, we recruit 100 participants who will receive a sensor. The number of participants is limited due to financial limitations for sensors and nasal swabs. These first 100 participants are referred to henceforth as the “sensor group”. As many requests were already made to the researchers for more spots to participate in the study even without sensors, an additional cohort is implemented for family, household members, or colleagues (in case the sensor will be placed at the workplace). Inclusion criteria remain the same.

### Recruitment

Recruitment started in March 2021 with the members of the OK Lab Cologne as a pilot run to enable changes if required. Following the 2-week trial, the recruitment of citizens started via a snowball system.

### Questionnaires

We discussed questionnaires with the members of the OK Lab Cologne and the Cologne School of AI during several workshops, and 18 questionnaires were pre-tested. As a result of these workshops, we modified 12 questionnaires. The remaining six questionnaires were pre-validated standardized questionnaires that could not be changed. Participants receive a weekly health questionnaire to report symptoms of respiratory infections, including COVID-19. If someone reports an infection, they automatically receive a detailed symptom questionnaire. It is also possible to report an acute infection with a so-called spontaneous reporting form. We will use parts of the further questionnaires to adjust the statistical analyses for confounding variables and to assess risk factors commonly associated with respiratory infections, such as sociodemographic variables, preexisting medical conditions, stress, workload, exercise, quality of life, vaccinations, and travel. Due to the current pandemic, we also included specific questionnaires about the preventive measures against COVID-19, such as hygienic measures and contacts. For assessment of the usability of PIA, we provide a technology readiness questionnaire 30 days after the individual starting time [46] as well as an usability questionnaire 90 days after the start [47]. All questionnaires remain accessible in-app after they have been completed, in case the participants may want to use them for their documentation purposes.

### Nasal swabs

Each participant in the sensor group is given the opportunity to collect a nasal swab at home. Participants can send the swab by using a provided pre-paid box to the Institute for Virology of the Hannover Medical School (MHH) at no cost. Each swab is tested by MHH for viruses such as influenza A and B, parainfluenza 1 to 4, rhinovirus, human metapneumovirus, adenovirus, respiratory syncytial virus (RSV), four endemic human coronaviruses (229E, NL63, HKU1, OC43), and SARS-CoV-2 via multiplex PCR. The results of those tests are provided in-app for the participants individually in their own personal accounts. Any infections with influenza or SARS-CoV-2 have to be reported to the health department of Cologne according to the §6 of the Law of Preventing Infections (IfSG) [48].

### Statistical analysis

Due to the small sample size as a pilot and feasibility study, we will focus statistics on descriptive methods. Hence, we did not perform a power calculation. Where feasible, we will pool data from SMARAGD with data from similar studies we conducted to gain greater statistical relevance and comparison between different cohorts. Regarding feasibility, we define success as follows: (1) 75% of participants fill in weekly questionnaires on health status at least every other week on average, (2) at least 50% all other questionnaires are submitted; (3) missing items within submitted questionnaires are less than 10%, (4) 75% of nasal swabs are sent for laboratory analysis, and (5) 75% of sensors collect data during 75% of time of deployment.

### Discussion

To our knowledge, we set up the first study that links air pollution with regularly, digitally reported health data that uses low-cost sensors which provide scientifically acceptable data quality.

### SMARAGD and the benefits of citizen science for epidemiology

SMARAGD allows researchers and citizens to work collaboratively on a specific project. As a result, the researchers learn how scientific approaches can be made more accessible and integrated into participants’ daily life. In our case, the citizens requested additional questionnaires regarding sleep patterns and workload. We also integrated a combination of weekly and monthly as opposed to daily questionnaires on health status, which were originally proposed by the epidemiologists. Hence, we added questionnaires on topics of interest to the participants or their preferred structure respectively that would otherwise have been ignored. After deciding which questionnaires and topics to use, we tested questionnaires via on-site and online cognitive pretests [49]. During these tests, many requests for changes to the questionnaires were expressed by the citizens, which we not only implemented within SMARAGD but will also take into consideration in future studies. However, for some pre-validated questionnaires [46, 47, 50–52], no changes were possible as otherwise the possibility of a comparison with other studies would no longer be possible and the validation would no longer apply. This led to critical discussions between researchers and citizens about the use of those questionnaires, and concerns were raised regarding comprehensibility of the questionnaires themselves. If citizens deem phrases unintelligible, the benefits of validated tools may need to be reconsidered. Working with citizens in the project also allows addition of the citizens’ (sometimes highly specialized) expertise which should be taken into account. In SMARAGD, we used this specific expertise of our citizen partners for the development of the low-cost air pollution sensors. In addition, participants with a certain area of expertise can aid recruitment among acquaintances via a snowball system. The use of citizen science enables scientists and research institutes to focus on the issues that the public cares about. The desire to determine particulate matter levels led to the creation of initiatives, where citizens operate particulate matter sensors and feed the data into a database [53]. Other civil society movements on climate change such as “Fridays for Future” are fighting for the right to a healthy future and for politicians to acknowledge the need for a livable world in the decades to come [54]. Scientists see the same need, creating visibility for urgent needs such as climate change [55]. These movements are examples of society wanting its views and needs recognized. In Germany, politicians have reacted by planning to introduce citizens’ assemblies. They will meet on specific issues and develop recommendations for action to be considered by the federal government in Germany [56]. With the SARS-Cov-2 pandemic, people also started to recognize knowledge on health topics as urgent and valuable. Society learned about the meaning of concepts such as incidence, the reproduction number (R0), different kinds of vaccination, and ways to protect oneself based on scientific results. Scientific television formats [57] or podcasts about the coronavirus pandemic with renowned epidemiologists and virologist [58] became popular. All these phenomena demonstrate that citizens want to engage in topics that concern them. Citizen science can also support and leverage such needs by providing access to high-quality scientific information or by fostering the ability to think scientifically when engaging in a critical discourse. It also creates a connection between nature, environment, and the citizen scientists’ own health and well-being [10]. The project SMARAGD takes these demands for participation into account and offers inclusion in areas like engineering (sensors), digital matters and information technology (PIA), environment (air pollution), and health (respiratory infections) in one project. Both citizen groups who are part of the project existed before and worked with local pollution data. SMARAGD is just one project among others on which they have worked. Therefore, the lessons learned from the project will not only benefit the researchers for future projects but also may benefit the citizens for their future engagement in other projects.

Citizen science as an approach can also be used to counteract anti-scientific worldviews by engaging citizens in science and showing them how scientists come to their conclusions. This provides a possibility of changing opinions and values and of providing people with access to influence policies and politics [10, 13]. This is especially important in current times, where misinformation is prevalent. Experiencing and contributing to science may be a way to show that scientists usually aim to work for the benefit of the public. Whether this is something that SMARAGD can also convey will likely be assessable in project evaluations.

For epidemiology, it is described that those affected by something should be involved in its respective research [59]. German Medical Ethicist Alena Buyx remarked: “Every participant is a PI” [60]. This lays the foundation for citizen science in epidemiology. Nevertheless, only very few projects in the field of health and medicine have adopted the approach, e.g., in May 2022, only 30 projects out of 230 on the EU-citizen science platform are tagged as health and medicine projects [61].

### Limitations of citizen science in epidemiology

Citizen science also comes with challenges. Motivating citizens to participate in projects in their spare time, just to gain knowledge and understanding, may be difficult. Generally, citizens engage in science due to personal interests or because they want to contribute to something meaningful [62–65]. However, these reasons may change over time, which is in contrast to the timeline of many epidemiological projects that take years from conceptualization to publication and therefore require long-term commitment. Relevant participation in projects, i.e., from start to the end, may often take longer than anticipated at the beginning and necessitate perseverance from people who do this as a leisure time activity. With SMARAGD, we intend to keep motivation high through meetings between citizens and researchers, even if these have, to date, mostly been possible only online due to the coronavirus pandemic. Whether these meetings serve the purpose of maintaining sufficient continuous engagement will only become clear at the end of the project.

From a scientist’s perspective, one of the benefits of citizen science is to gain different opinions from multiple backgrounds, e.g., regarding intelligibility of questionnaires. However, this can be challenging because time and effort must often be invested in communication between researchers and citizens [10, 13]. In addition, citizens may be interested in other topics than researchers. For example, for SMARAGD, one of the longer discussions was which questionnaires to include or exclude. As researchers from the field of infectious disease epidemiology, we focused our attention strongly on the then-emerging COVID-19 pandemic. The scientific team therefore wanted to include questionnaires on contacts, hygienic measures, and the like. However, the citizen group was more interested in allergies, which were perceived to have a greater affect in their daily life over the long term. Compromises pertaining to the scope of questionnaires had to be found to suit both sides. For SMARAGD, questionnaires about hygienic measures were implemented less often than intended because of the citizens’ lack of specific interest while including basic questions on allergies, which were not originally conceived in the study scope as defined by the scientists.

Concerns regarding data security may make it difficult to recruit study participants in Germany [66]. This might also be applied to citizen science approaches. As a countermeasure, we specifically offered discussions regarding data security resulting in a traditional epidemiological approach with questionnaire and medical data being pseudonymized and data primarily accessible only to researchers who are bound by data privacy regulations and practices.

Another factor that could lead to a general problem in citizen science is the fact that citizens invest their spare time in the project, whereas researchers are usually paid, even though both parties may spend an equal amount of resources [15, 16]. So far, this has not been an issue between citizens and researchers in SMARAGD. However, in the first workshop, we jointly decided that the major part of communication and study organization should remain with the researchers, rather than be implemented as shared tasks. Therefore, the majority of the overall work rests with the researchers. This asymmetry is reflected in the fact that our citizen partners are not among the authors of this paper.

Probably the most well-known challenge of citizen science is that specific groups of people are more likely to engage in specific projects. Therefore, it can be argued that projects using a citizen science approach do not represent the general population and therefore might be biased [10, 67]. In the case of SMARAGD, we expect a disproportional number of young- to middle-aged men, with relatively good health and higher education, as this was the composition of the preexisting group (OK Lab Cologne) supporting the project. However, this does not always have to be considered a challenge or even a problem; especially for causal research aims, the composition of the population does not necessarily have an impact [68] which applies to the exploration of the relation between air pollutants and health indicators in SMARAGD. Whereas, e.g., for surveys that aim at describing characteristics of a certain population, representativeness is essential [68]. In SMARAGD, we assess feasibility of a specific study type in a convenience sample, i.e., citizen scientists, without deducing a general rule for all of epidemiology. Another objective of SMARAGD is to compare measurements of low-cost sensors to those from high-quality reference instrumentation; therefore, the selection of participants needs to be described but does not necessarily affect parts of the research done in SMARAGD. However, an awareness of any bias is undoubtedly always required, but this applies to all population-based epidemiological research, not only citizen science. On the contrary, citizen science may even offer the chance to include groups into science that are otherwise possibly left out [10, 67].

One challenge that is still fraught with great uncertainty in citizen science projects is data protection [69]. If citizens engage deeply in scientific projects, they might encounter the collected raw data. In Germany, there are regulations in place considering data protection like the General Data Protection Regulations (GDPR) that provide the respective framework [70]. In particular, medical data, as stated in §9 of GDPR are considered highly sensitive and are protected accordingly [70]. The GDPR or guidelines like the “Good Epidemiological Practice” [59, 70] do not consider the involvement of nonscientific personnel who have not signed any agreement with data protection regulations. The “Leitfaden für rechtliche Fragestellungen in Citizen-Science-Projekten [Guide to Legal Issues in Citizen Science Projects]” provides some information on the GDPR in the context of citizen science [69] but is considered as an overview rather than a specific and concrete guideline. For example, if participants get to know each other in workshops, they might recognize each other’s provided data sets, even if answer categories are grouped providing less detailed information on individuals while at the same time loosing information that might be of scientific interest. In a small project like SMARAGD, recognition can easily occur, especially because many of the participants have known each other for a long time.

**Fig. 2:**
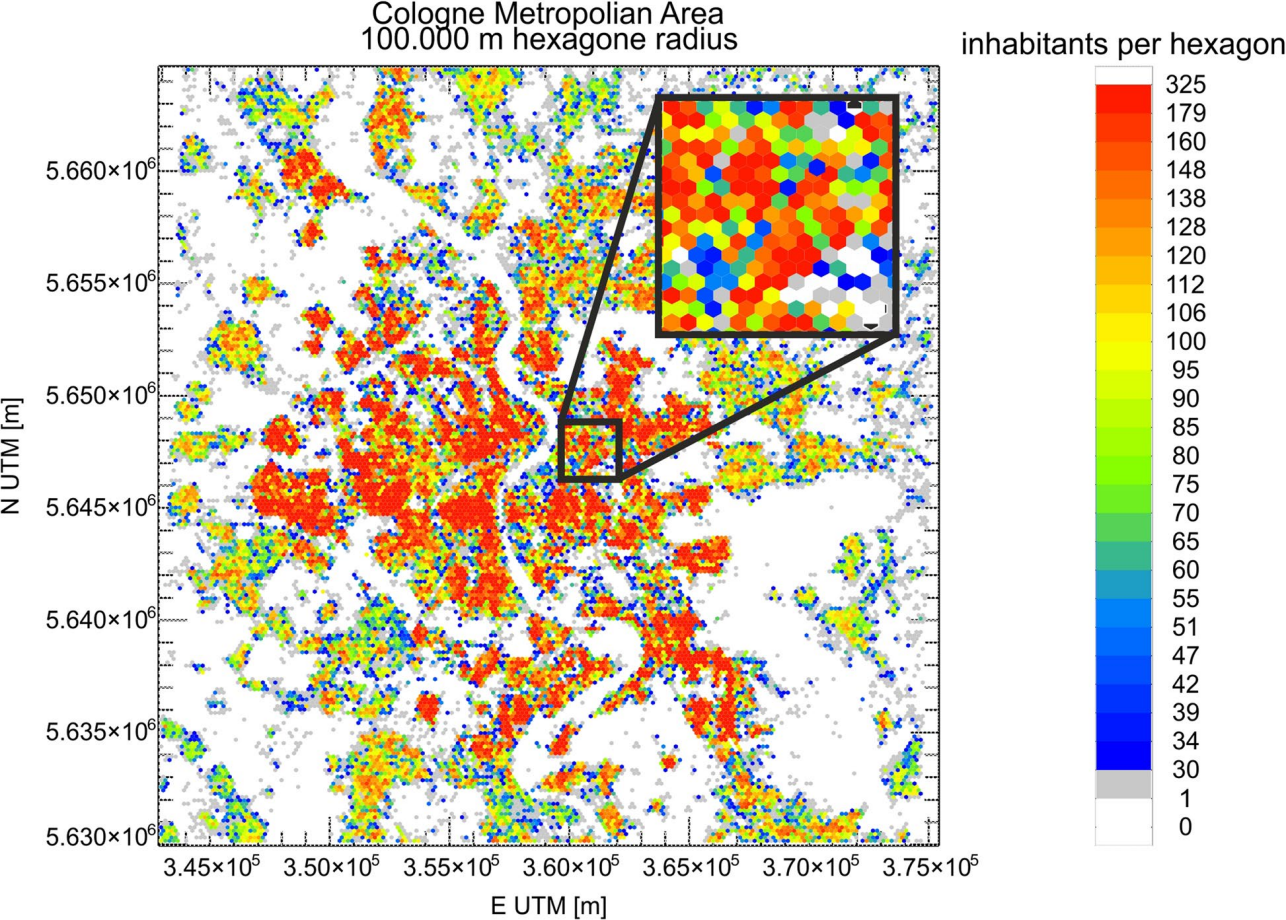
Cologne Metropolitan Area with 100-m hexagon radius showing the number of inhabitants per hexagon. Data from https://dataforgood.fb.com/docs/high-resolution-population-density-maps-demographic-estimates-documentation/#datasets downloaded on 2021/04/18

One specific data protection issue is the availability of the sensor location data to all participants during the data collection period as the place of residence or workplace is one of the main aspects in the project. Therefore, we had to find a suitable way to collect this information and provide them to participants without violating the GDPR. Therefore, we superimposed a hexagon pattern over the city of Cologne, which is structured in a way that usually more than 30 people live in one hexagon (Fig. 2). If fewer than the expected 30 individuals live in one hexagon, data is represented in an adjacent hexagon. This allows for the study of spatial patterns but ensures that housing locations of participants remain confidential. Each sensor is sorted into the respective area by researchers who have access to address data but not to medical data and who have signed a data privacy notice. Participants will be able to see information regarding air pollutants of the sensors online but will not be able to identify the address of a specific sensor, only its approximate location within the city.

The fact that participants see the data set might also conflict with the individual’s right not to know about conditions or results of any medical measure. Participants can indicate in the consent form that they do not want to be informed about the result of the nasal swab laboratory analysis. However, they might identify themselves and their result in the data set. Therefore, we decided that any combination of identifiable answers that occurs less than five times will be recategorized in a less detailed manner so that no identification can take place. In general, citizen science approaches are easier to apply for noncritical topics. In SMARAGD, we chose not to include, e.g., questions on sexual contacts, although these are possibly associated with the risk of infections. The new “Citizen Science Weißbuch (Citizen Science White Paper)” in Germany also recognizes the great uncertainty regarding data protection in citizen science; however, it too does not offer a ready-to-use solution regarding data privacy [71]. Still, for the provision of the data set to the citizen, all these data protection aspects must be considered.

### Conclusion

Citizen science can make research more meaningful by including thoughts and opinions of those affected. Bringing researchers and citizens together allows knowledge transfer from both ends so that everyone benefits from science. It also allows civil engagement which many people demand. Therefore, citizen science can be a way to take science onto a new level. Even though citizen science brings many benefits, it also bears its challenges. Long-term motivation of citizens to participate and communication barriers between researchers and citizens are among the more general limitations. Other challenges may arise, particularly in epidemiological studies, such as data protection issues due to working with medical data or selection bias. Real-world projects such as SMARAGD are needed to assess the specific benefits and develop how to overcome the challenges.

## Data Availability

Not applicable.

## Abbreviations

AI: Artificial intelligence
EURAD: European Air Pollution Dispersion
FZJ: Forschungszentrum Jülich
GDPR: General Data Protection Regulation
HMGU: Helmholtz Centre Munich
HZI: Helmholtz Centre for Infection Research
MHH: Hannover Medical School
OK Lab: Open Knowledge Lab
PI: Principal investigator
PIA: Prospective Management and Monitoring App
SMARAGD: Sensors for Measuring Aerosols and ReActive Gases to Deduce health effects
